# Investigation on the influence of ultrasonic pretreatment on color, quality and antioxidant attributes of microwave dried Inula *viscosa* (L.)

**DOI:** 10.1016/j.ultsonch.2022.106184

**Published:** 2022-09-27

**Authors:** Alev Yüksel Aydar, Tuba Aydın, Tuncay Yılmaz, Anjinelyulu Kothakota, Socol Claudia Terezia, Criste Florin Leontin, R. Pandiselvam

**Affiliations:** a Department of Food Engineering, Manisa Celal Bayar University, Turkiye; b Agro-Processing & Technology Division, CSIR-National Institute for Interdisciplinary Science and Technology (NIIST), Trivandrum, Kerala 695 019, India; c Department of Genetics, University of Oradea, Oradea 410048, Romania; d Physiology, Biochemistry and Post-Harvest Technology Division, ICAR-Central Plantation Crops Research Institute (CPCRI), Kasaragod, Kerala 671124, India

**Keywords:** Inula *viscosa* (L.), Microwave drying, Ultrasound, Antioxidants, Total phenols

## Abstract

Impact of various ultrasound pretreatment and microwave drying parameters on the qualitative and antioxidant characteristics of Inula viscosa (L.) was investigated in this study. The leaves of Inula viscosa (L.) were sonicated for 10, 20, and 30 min in an ultrasonic bath (37 kHz, 150 Watts). Microwave drying was done at three distinct times (1, 3, and 5 min) and with three different microwave power levels (100, 180, and 300 Watts). Microwave dried samples were tested for color characteristics (L*, a*, b*), chlorophyll, carotenoid, total phenol, and antioxidant content. All dried samples were prepared by infusing them in hot water as tea, and the sensorial properties of teas including odor, color, aroma, and overall acceptability were evaluated by panelists. For 10, 20, and 30 min of ultrasound pretreatment, the L* values of leaves varied from 37.70 to 49.76, 34.97 to 46.25, and 27.88 to 43.34, respectively. The total carotenoid concentration ranged from 0.12 to 0.32 mg/g DW, while the total chlorophyll content was from 0.44 to 0.94 mg/g DW. The antioxidant activity of Inula viscosa (L.) leaves that were dried at 300 Watts for 5 min did not change significantly as a result of ultrasound pretreatment. There was a significant positive correlation between aroma and TPC, as well as between color and overall acceptability. The darkest-colored teas were deemed preferable by the panelists.

## Highlights

• Samples dried at 100 and 180 W had higher chlorophyll and carotenoid levels than samples dried at 300 W.
• A positive correlation was found between color and overall acceptability (r = 0.77)
• The lower DPPH of the *Inula viscosa L.* extracts obtained when the ultrasound pretreatment time was lower than 20 min.
• Inula viscosa L. leaves subjected to 10 and 20 min of ultrasound had slightly higher L* values than those exposed to 30 min of sonication.

## 1 Introduction

The Mediterranean plant Inula (syn. Dittrichia) *viscosa* (L.) is widely utilized in traditional medicine. The herbaceous perennial plant, commonly known as Inula, belongs to the Asteraceae family and is distinguished by its sticky leaves and yellow blooms [1]. Inula, which produces unifloral honey in Europe, is a low-cost, effective pesticide for managing varroosis in Apis mellifera colonies [2], and it also appears to have a part in the cycle of auxiliary insects that control *Bactroceraoleae*, one of the most common pests of olive trees [3]. *I. viscosa's* aerial portions include triterpenoids in the form of free alcohols, acetates, and fatty esters. Leaf extracts contain more oxygenated sesquiterpenes and flavonoids that are responsible for antioxidant activities [1]. Several researchers have also found that Inula *viscosa* (L.) has antibacterial, antipyretic, and anti-inflammatory properties. This is because this plant has many physiologically active components, such as flavonol derivatives and phytochemicals such as polyphenols and sesquiterpenes [2], [4], [5].

In the food industry, drying is a popular procedure for producing and storing food products with a longer shelf life [6]. Pretreatments such as ultrasound, which retain or reduce the quality loss of foods to a minimum, are applied before drying since the drying process might generate negative consequences for the final product's quality characteristics [7]. Ultrasound is one of the non-thermal technologies that has been increasingly popular in recent years in the food industry and minimizes internal resistance by opening microscopic pores in the food during drying [8], [9]. It is well recognized that the efficiency of ultrasonic extraction is directly influenced by acoustic cavitation, mechanical function, and thermal function, with cavitation being the most crucial element [10]. Additionally, ultrasound treatment enhances diffusion through the pores of the solid in the liquid phase surrounding the particles as well as heat and mass transfer [11]. Many foods, including apple, olive, carrot, garlic, okra, kiwi, and melon, have been subjected to ultrasound pretreatment before drying in literature. By decreasing the drying period, ultrasound was found to generate considerable increases in product quality parameters such as color, phenolic substance, and antioxidant compounds in several of these investigations [12], [13].

Several techniques to preserving food and plants are now being considered, particularly in light of advancing technology and changing nutritional habits [14]. People prefer and use practical and easy-to-implement applications, such as the microwave drying method, which stands out among these ways [15], [16], [17]. Microwave drying results in faster moisture transfer than traditional drying processes, and the nutritional value of food components is preserved better [18]. Although microwave drying has been applied to several plant and foods in the literature, no study has investigated the effect of ultrasound combined microwave treatment on this plant, which is consumed in dried form. Thus, the aims of this work were to investigate the effect of ultrasound pretreatment and microwave drying time and power on the quality of Inula *viscosa* (L.) leaves and identify suitable treatments for producing high‐quality dried leaves.

## 2 Material and methods

### 2.1 Inula *viscosa* (L.) *plant and chemicals*

It was delivered between the months of June and September 2021 from the provinces of Manisa and Izmir. The plants were put in vacuum-sealed packages, and they were brought to our laboratory. The leaves were maintained at refrigerator temperature (4 °C) until they were analyzed. The solvents including acetone, methanol, and DPPH (2,2-diphenyl-1-picrylhydrazyl) were purchased from Sigma-Aldrich (Steinheim, Germany). The gallic acid and Folin-Ciocalteu reagents were provided by Merck (Darmstadt, Germany). Analytical-grade solvents and reagents were employed in this study.

### 2.2 Ultrasound pretreatment and microwave drying

Ultrasound pretreatment was applied in an ultrasonic bath (Elmasonic S50 R, Germany, 5L, frequency of 37 kHz, power 150 Watts) and the ratio was adjusted to 1:5 (plant:water ratio). The Inula *viscosa* (L.) leaves were sonicated for 10, 20 and 30 min at room temperature during ultrasound did not exceed to 40 °C. A microwave oven (GE83X, Samsung, Turkey, 2450 MHz and 23 L capacity) was used for drying of leaves. About 5 g of samples were weighted and puton a glass drying tray (10 cm diameter). Then they were left to dry at 100, 180, and 300 W microwave power levels for 1, 3 and 5 min. The weight of samples was recorded after microwave drying was completed. The final moisture content of samples is calculated by the known initial moisture content of samples. The experiments were conducted in accordance with the experimental scheme shown in Table 1 and they were performed in triplicate.

**Table 1:** Treatment codes and parameters.

Treatment Code	Ultrasound time (min.)	Microwave Time (min.)	Microwave Power (Watts)
10–1-100	10	1	100
10–3-100	10	3	100
10–5-100	10	5	100
10–1-180	10	1	180
10–3-180	10	3	180
10–5-180	10	5	180
10–1-300	10	1	300
10–3-300	10	3	300
10–5-300	10	5	300
20–1-100	20	1	100
20–3-100	20	3	100
20–5-100	20	5	100
20–1-180	20	1	180
20–3-180	20	3	180
20–5-180	20	5	180
20–1-300	20	1	300
20–3-300	20	3	300
20–5-300	20	5	300
30–1-100	30	1	100
30–3-100	30	3	100
30–5-100	30	5	100
30–1-180	30	1	180
30–3-180	30	3	180
30–5-180	30	5	180
30–1-300	30	1	300
30–3-300	30	3	300
30–5-300	30	5	300

### 2.3 Dry matter (DM)

The oven drying method was used for determination of moisture content and dry matter. According to this method pesticide contaminated leaves before and after sonication were homogenized and dried at 120 °C until a constant weight [19]. The final moisture content of samples calculated from the known initial moisture content of samples. The dry matter (%) of samples was computed by subtracting the moisture content from 100 %.

### 2.4 Color analysis

The values of L* (lightness), a* (redness-greenness), and b* (yellowness-blueness) of leaves were measured in the CIE-LAB space with a Konica Minolta CR 300 Model, VA. Three replicate measurements were taken for each sample after the colorimeter was calibrated against a white and black surface. The average and standard deviation of the results were presented.

### 2.5 Chlorophyll and carotenoid contents

Total carotenoids, total chlorophylls, and chlorophyll *a* and b concentrations were measured as reported by Salachna et al. [20]. A homogenized sample of about 0.1 g was weighed and blended with 15 mL of 80 % acetone before being combined with 80 % acetone in a 50 mL volume measurement flask. For 5 min, the flask was placed in an ultrasonic cleaner. The solution was then transferred to a tube and centrifuged for 10 min at 10000 rpm and room temperature. The absorbance of the samples was measured against a blank sample (80 % acetone) at 441 nm, 646 nm, 652 nm, and 663 nm. The concentrations of chlorophyll *a*, chlorophyll *b*, total chlorophyll, and total carotenoids in the plant material were calculated by the equations below and given as mg/g DW in Fig. 2.

(2) Chlorophylla(µg/gDW)=(12.21×E663-2.81×E646)×(V/1000×m)

(3) $$\mathrm{Chlorophyll}\;b\;\left(\mu g/g\;DW\right)=\left(20.13\times E646-5.03\times E663\right)\times \left(V/1000\times m\right)$$

(4) $$\mathrm{Total}\;chlorophyll\;\left(\mu g/g\;DW\right)=\left(27.8\times E652\right)\times \left(V/1000\times m\right)$$

(5) $$\mathrm{Total}\;carotenoids\;\left(\mu g/g\;DW\right)=[\left(1000\times E441\right)-3.27\times (12.21\times E663-2.81\;\times E646)-104\times \left(20.13\times E646-5.03\times E663\right)]\times [V/1000\times \left(m\times 229\right)]$$

### 2.6 Total phenolic content (TPC) and antioxidant capacity (DPPH)

The products obtained through ultrasound-assisted extraction were utilized to calculate TPC using a Folin-Ciocalteu method [21]. To make a plant extract solution with a concentration of 1 mg/mL, a determined amount (10 mg) of dried extract was dissolved in 10 mL of ethanol (50 %). The reagents were prepared according to the instructions. 0.02 mL of plant extract solution was used in a multi-mode microplat reader (Thermo Scientific MultiskanGo Microplate Spectrophotometer, Model 1510, Vantaa, Finland), while gallic acid was used to determine the standard curve. Then, 0.08 mL Na_2_CO_3_ (7.5 %) and 0.1 mL Folin-Ciocalteu reagent (1:10) were added. For 30 min, the plate was kept in the dark at room temperature (25 °C). The absorbance of all the samples was measured at 765 nm after incubation.

The DPPH (2,2-diphenyl-1-picrylhydrazyl) assay was used to determine the free radical scavenging or antioxidant activity of extracts according to a published procedure [9]. The extract had a concentration of 1 mg/mL in 50 % ethanol. According to the procedure, the analysis was performed on microplate reader. In the microplate reader, 10 µl (0.01 mL) of plant extract solution was added in each well of the microplate, and then 90 µl (0.09 mL) deionized water was added. In a darkened room, 100 μL (0.1 mL) of DPPH solution was added to each plate. The plate was incubated in the dark for 30 min at 25 °C ambient temperature. The same process was used to make a blank solution, but instead of plant extract, 50 % ethanol was used. The absorbance of all the samples was measured at 517 nm after they had been incubated. The activity of radical scavenging was measured as a percentage of inhibition.

### 2.7 Sensorial evaluation of tea samples

Sensory properties of teas prepared by drying Inula *viscosa* (L.) were determined by 10 panelists. 3 g of dried Inula *viscosa* (L.) leaves were infused with 300 mL of 70 °C distilled water in a tea pot. The tea samples were brewed for 2 min and, while it brewed, the pot was swirled five times. The tea was poured through a porcelain strainer and served in white porcelain teacups. The International Organization for Standardization [21] suggested the white porcelain tea wares to maintain a consistent result.The extract solution was filtrated and then used for sensory evaluation. Each tea sample was rated for its sensory properties, including color, aroma, odor, and overall acceptability, on a scale of 1 to 5 (for acceptability; 5 = like; 4 = like slightly; 3 = neither like nor dislike; 2 = dislike slightly; 1 = dislike, for other properties; 1 = very weak; 2 = fairly weak; 3 = neither weak nor strong; 4 = fairly strong; and 5 = very strong).

### 2.8 Statistical analysis

Using SAS 9.2. (SAS Institute Inc., Cary, NC, USA) data were analyzed by ANOVA to determine the significance of the effects of the factorswith p < 0.05 indicating that the effects were significant. Tukey's honestly significant differences (HSD) test (α = 0.05) was used for post-hoc analysis. In this study, microwave power, time\ and ultrasound time were selected as independent factors, and each factor had three levels. The response factors included color properties (L*, a*, and b*), TPC, DPPH activity, total carotenoids, total chlorophylls, chlorophyll_A_, and chlorophyll_B_. The design of the experiment was created with the help of Design Expert software (Version 11, Stat-Ease, Inc., Minneapolis, MN, USA), and the model fitness was evaluated by evaluating the coefficient of determination (R^2^), lack of fitness model, ANOVA for a linear model and F-value for each response software. The experiment included a total of 27 run factorial points, as demonstrated in Table 1. The correlation between sensorial attributes of Inula *viscosa* (L.) teas and the physicochemical properties of the leaves was determined by the Pearson correlation coefficient (Table 4).

## 3 Results and discussion

### 3.1 The effect of ultrasound and microwave processing parameters on dry matter

**Fig. 1:**
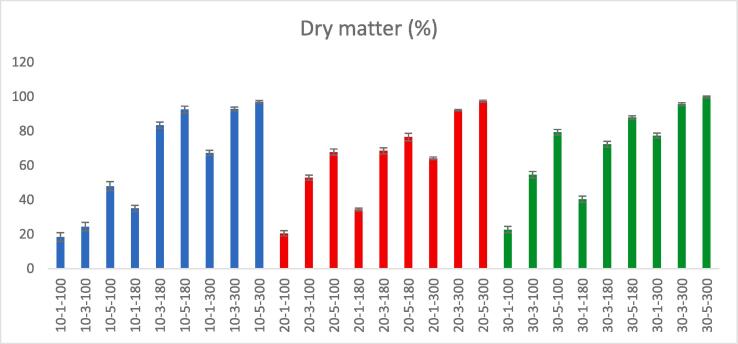
Changes in dry matter of samples after different drying parameters.

Moisture content was determined by keeping Inula *viscosa* (L.) at 105 °C until the plant weight became constant. Ash content was analyzed by using a muffle furnace to heat the 5 g of Inula *viscosa* (L.) plant at 550 °C for 5 h. Fig. 1 depicts the changes in dry matter (%) of samples dried at various drying parameters. According to ANOVA results, microwave time and power have significant effect on dry matter (p < 0.001), while no significant effect was found between ultrasound pretreatment and the dry matter of leaves (p > 0.05). The moisture content was found to decrease linearly as microwave power and time were increased. As a result, samples dried at 300 Watts had the highest dry matter percent. It can be explained that due to the penetration of microwave energy into the substance and the creation of a large difference in vapor pressure between the substance's interior and exterior, the use of microwaves reduces the drying time and increases the dry matter [17], [22].

**Fig. 2:**
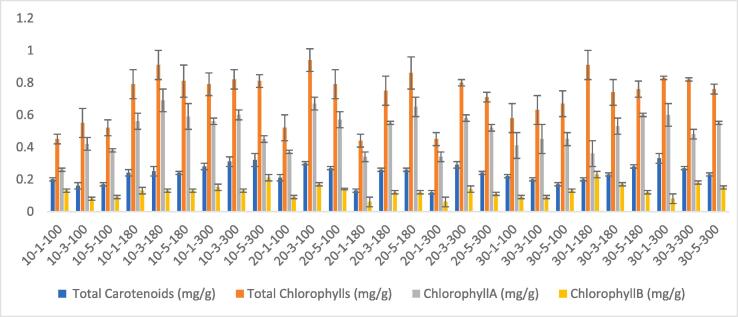
Changes in total carotenoids, chlorophylls, chlorophyl_a_ and chlorophyll_b_ content.

### 3.2 The effect of ultrasound and microwave processing parameters on color

The pretreatments used before drying as well as the drying conditions have an impact on the quality of a dehydrated product [23]. It is well established that the quick generation of heat in microwave applications has made it possible to save time and improve final product quality (increased flavor, color, and nutritional content) [24]. Color (L *, a *, b *) parameters were measured with Konica Minolta, Chroma Meter CR −5 model and the change in color properties is shown in Table 2. The lightness (L*) of the samples ranged from 27.88 to 49.76 and the a* values ranged between 16.95 and 26.96. Samples subjected to 10 and 20 min of ultrasound had slightly higher L* values than those exposed to 30 min of sonication. The higher b* values were observed in samples 10–1-100 and 20–1-100 than the other samples, which were 27.47 and 26.96, respectively, while the samples obtainedby 30 min of ultrasound pretreatment and drying for 3 min of microwave at 300 Watts showed the lowest L* value (darker) and lowest b* values. The values of a* were found in the green zone, and the variationwas important among the treatments where there is a statistically significant difference. In our study, highest a* value was −2.99 in the treatment of 10–5-180, and it was lowest at −9.93 in 20–1-100 samples.

**Table 2:** Changes in color parameters, TPC and DPPH values at different treatments.

Treatment	L*	a*	b*	TPC (mg/g DW)	DPPH (%)
10–1-100	41.98 ± 1.53^abcd^	−7.37 ± 0.49^bcdefg^	27.47 ± 1.06 ^ab^	34.96 ± 3.76^bcde^	41.07 ± 4.60^jk^
10–3-100	43.57 ± 1.82^abcd^	−7.99 ± 0.43^cdefg^	22.20 ± 1.17^cde^	22.77 ± 2.15^fgh^	32.14 ± 1.64^kl^
10–5-100	42.25 ± 5.12^abcd^	−4.77 ± 0.92^abcd^	23.18 ± 2.40^abcde^	21.19 ± 0.85^gh^	69.85 ± 0.70^def^
10–1-180	41.94 ± 2.50^abcd^	−9.54 ± 0.74 ^fg^	21.60 ± 2.01^cdef^	22.59 ± 1.12^fgh^	29.11 ± 4.21 ^l^
10–3-180	37.70 ± 2.34^bcde^	−6.57 ± 1.08^abcdefg^	20.47 ± 0.53^cdefg^	42.89 ± 0.17^b^	95.04 ± 0.47^a^
10–5-180	47.16 ± 4.95^ab^	−2.99 ± 1.49 ^a^	20.01 ± 0.59^cdefg^	29.58 ± 0.84^defg^	80.26 ± 0.31^bc^
10–1-300	39.67 ± 6.89^abcde^	−4.09 ± 1.89^abc^	20.87 ± 0.92^cdefg^	38.87 ± 2.03^bc^	77.40 ± 5.15 ^cd^
10–3-300	49.76 ± 3.42^a^	−3.79 ± 1.56 ^ab^	24.25 ± 0.76^abc^	36.81 ± 1.70^bcde^	95.87 ± 1.79^a^
10–5-300	44.71 ± 1,34^abc^	−4.59 ± 1,42^abc^	23.41 ± 0,86^abcd^	34.43 ± 2.93^bcde^	96.69 ± 1.09^a^
20–1-100	46.25 ± 2.71^abc^	−9.93 ± 0.46 ^g^	26.96 ± 2.00^a^	37.62 ± 2.94^bcd^	35.50 ± 1.87^kl^
20–3-100	34.97 ± 2.31^cde^	−6.95 ± 1.11^bcdefg^	20.82 ± 1.07^cdefg^	22.43 ± 3.78^fgh^	34.95 ± 2.03^kl^
20–5-100	39.74 ± 8.88^abcde^	−4.62 ± 3.08^abc^	22.87 ± 3.14^bcde^	21.69 ± 1.93^gh^	49.23 ± 3.04^ij^
20–1-180	40.69 ± 2.50^abcd^	−7.88 ± 0.95^cdefg^	22.20 ± 0.20^cde^	22.33 ± 4.39^gh^	31.86 ± 0.16^kl^
20–3-180	35.20 ± 1.36^cde^	−5.57 ± 1.19^abcde^	18.98 ± 2.79^defg^	24.31 ± 2.30^fgh^	64.28 ± 0.78^efg^
20–5-180	44.06 ± 2.20^abcd^	−8.54 ± 1.31^defg^	21.75 ± 0.68^cdef^	21.98 ± 2.79^gh^	67.70 ± 0.16^defg^
20–1-300	43.16 ± 3.57^abcd^	−7.84 ± 0.77^cdefg^	23.87 ± 2.89^abcs^	28.29 ± 0.78^efg^	90.24 ± 2.42^ab^
20–3-300	44.33 ± 2.31^abcd^	−4.82 ± 1.26^abcd^	22.01 ± 0.96^cde^	21.23 ± 0.94^gh^	72.60 ± 1.01^cde^
20–5-300	42.32 ± 2.27^abcd^	−4.85 ± 0.78^abcd^	21.66 ± 1.56^cdef^	22.48 ± 0.88^fgh^	96.36 ± 0.94^a^
30–1-100	35.17 ± 3.23^cde^	−9.20 ± 1.13^efg^	22.30 ± 1.62^cde^	35.88 ± 1.07^bcde^	34.73 ± 4.21^kl^
30–3-100	41.75 ± 2.55^abcd^	−5.07 ± 1.28^abcd^	21.35 ± 1.04^cdefg^	22.60 ± 1.95^fgh^	52.65 ± 2.73^hi^
30–5-100	37.35 ± 1.83^bcde^	−7.62 ± 1.36^bcdefg^	21.84 ± 1.20^cdef^	19.21 ± 0.64 ^h^	81.75 ± 1.48^bc^
30–1-180	41.20 ± 1.52^abcd^	−7.91 ± 1.66^cdefg^	21.97 ± 0.71^cde^	22.13 ± 1.21^gh^	58.99 ± 5.46^ghi^
30–3-180	36.85 ± 5.30^bcde^	−7.63 ± 0.87^bcdefg^	19.30 ± 1.62^defg^	30.98 ± 0.90^cdef^	94.16 ± 0.78^a^
30–5-180	43.52 ± 1.75^abcd^	−7.62 ± 0.75^bcdefg^	22.74 ± 0.62^bcde^	42.25 ± 3.01^b^	94.98 ± 1.17^a^
30–1-300	32.56 ± 0.37 ^de^	−6.61 ± 0.93^abcdefg^	18.47 ± 0.58^efg^	25.42 ± 0.02^fgh^	94.43 ± 2.57^a^
30–3-300	27.88 ± 4.47 ^e^	−6.84 ± 0.94^abcdefg^	16.95 ± 1.16 ^fg^	39.01 ± 0.51^bc^	94.05 ± 3.59^a^
30–5-300	43.34 ± 6.92^abcd^	−5.83 ± 0.44^abcdef^	20.82 ± 1.82^cdefg^	54.39 ± 1.28 ^a^	96.53 ± 1.01^a^

### 3.3 The effect of ultrasound and microwave processing parameters on chlorophyll and carotenoids

The color changes were linked to a decrease in the ratio of chlorophylls to carotenoids, as well as the fact that both the carotenoid and, more importantly, the chlorophyll content decreases during the ripening process [25]. Heat, oxygen, light, and enzymes can all influence carotenoids, which are color pigments that give fruits and vegetables their unique orange color [23]. Total carotenoid and chlorophyll content in the fresh Inula *viscosa* (L.) was calculated as 0.43 mg/g DW and 1.12 mg/g DW, respectively. According to Table 4, total chlorophyll content was significantly correlated with total carotenoid (r = 0.77) and chloropyll_A_ (0.81) and chloropyll_B_ (r = 0.68). After distinct drying procedures, total carotenoid levels varied from 0.12 to 0.32 mg/g DW, while total chlorophyll content varied from 0.44 to 0.94 mg/g DW. The chlorophyll and carotenoid content of samples dried at 100 and 180 Watts was slightly higher than that of those dried at 300 Watts. Similar to chlorophyll and carotenoid levels, chlorophyl a and chlorophyl b levels were also lower in samples dried at the highest power level for a long time. There is a positive correlation was found between total carotenoids and chlorophyll_A_ (0.72).

### 3.4 The effect of ultrasound and microwave processing parameters on total phenolic content and antioxidant scavenging activity

The results of TPC are given in Table 2. The highest and lowest TPC were obtained after 30 min ultrasound pretreatment. The maximum total phenolic content found was 54.39 ± 1.28 mg GAE/g at 300 W for 1 min drying, and the minimum content observed was 19.21 ± 0.64 mg GAE/g at 300 W for 5 min drying. By enhancing molecular interactions between the electromagnetic field and the sample, increasing microwave power can improve extraction efficiency. Longer microwave radiation, on the other hand, may cause some phenolic compounds to be destroyed [26]

The percentage DPPH radical scavenging activity values of *I. viscosa*obtained by different drying parameters is displayed in Table 2. The highest anti-radical activity was 96.53 ± 1.01 % (30–5-300) and the least was 29.11 ± 4.21 % (10–1-180). Except for the DPPH, no model was well fitted to all responses since the lack of fit p value was significant (p < 0.05) and model p values were greater than 0.05. Table 3 shows that the DPPH activity was significantly influenced by the linear effect of ultrasound time (A), microwave time (B) and microwave power (C). An analysis of the effect of the independent factors on DPPH showed the significant variation in DPPH% by varying the time and power during the drying, and the model was significant with a R^2^ value of 0.8899. The P-value of model <0.05 and P-value of lack of fit higher than 0.05 also confirmed that the model is significant. DPPH activity of Inula *viscosa* (L.) increased as microwave time and power increased (Fig. 4). This could be because microwaves improve extraction by increasing the internal pressure of solid medium; as a result, phenolic and antioxidant chemicals can be leached in less time using microwaves than with typical extraction methods [27]. The lower DPPH of Inula *viscosa* (L.) extracts were obtained when the ultrasound pretreatment time was lower than 20 min. The regression equation for DPPH while considering the significant terms is given below:

(6) $$DPPH\left(\%\right)=-18.04226+0.791125A+6.68688B+0.256842C$$

**Table 3:** ANOVA table for DPPH.

Source	Sum of Squares	F-value	p-value
**Model**	7307.48	29.62	< 0.0001
A-Ultrasound time	500.70	6.09	0.0313
B-Microwave time	1430.86	17.40	0.0016
C-Microwave power	5375.92	65.38	< 0.0001
**Residual**	904.49		
Lack of Fit	869.37	5.50	0.1632
Pure Error	35.11		
**Cor Total**	8211.97		
Mean vs Total	66957.65		
**Linear vs Mean**	**7307.48**	**29.62**	**< 0.0001**
2FI vs Linear	260.07	1.08	0.4123
Quadratic vs 2FI	21.54	0.0576	0.9799
Cubic vs Quadratic	587.76	11.16	0.0834
C.V. %	13.57		
R^2^	0.8899		
Adj R^2^	0.8598		
Pred R^2^	0.7632		
Adeq Precision	16.6821

**Table 4:** Correlation coefficients between different parameters of Inula *viscosa* (L.).

Parameters	Total Car. (µg/g)	Total Chl. (µg/g)	Chl. A (µg/g)	Chl.B (µg/g)	Color	Aroma	Odor	Overall acceptability	Dpph (%)	TPC (mg GAE/g DW)
Total Car. (µg/g)	1									
Total Chl.(µg/g)	**0.77**	1								
Chl. A(µg/g)	**0.72**	**0.81**	1							
Chl. B (µg/g)	0.47	**0.68**	0.21	1						
Color	−0.20	−0.29	−0.25	−0.25	1					
Aroma	−0.15	−0.08	−0.17	−0.08	0.41	1				
Odor	0.43	0.39	0.29	0.24	0.19	−0.17	1			
Overall acceptability	0,13	−0.23	−0.23	−0.04	**0.77**	0.33	0.30	1		
Dpph (%)	0,36	0.37	0.36	0.25	−0.02	−0.39	0.36	−0.11	1	
TPC (mg GAE/g DW)	0,23	0.08	0.11	0.18	−0.06	**0.91**	0.28	−0.01	0.40	1

The coefficients written in bold are significant (p < 0.05).

**Fig. 3:**
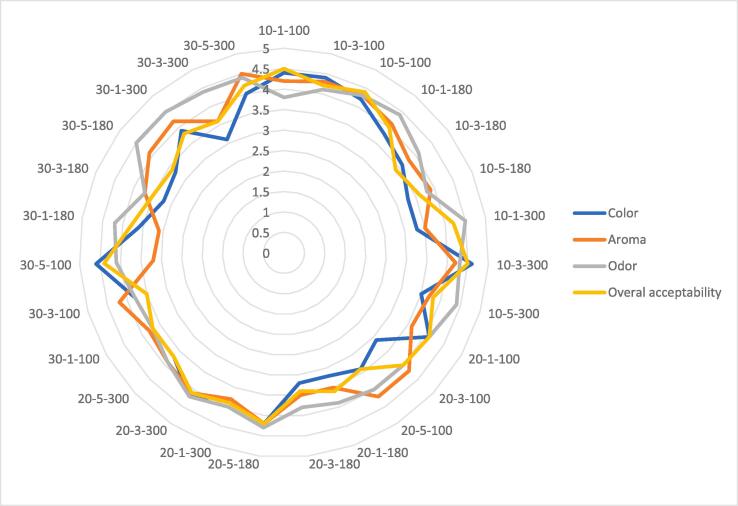
Sensory properties of Inula *viscosa* (L.) teas.

**Fig. 4:**
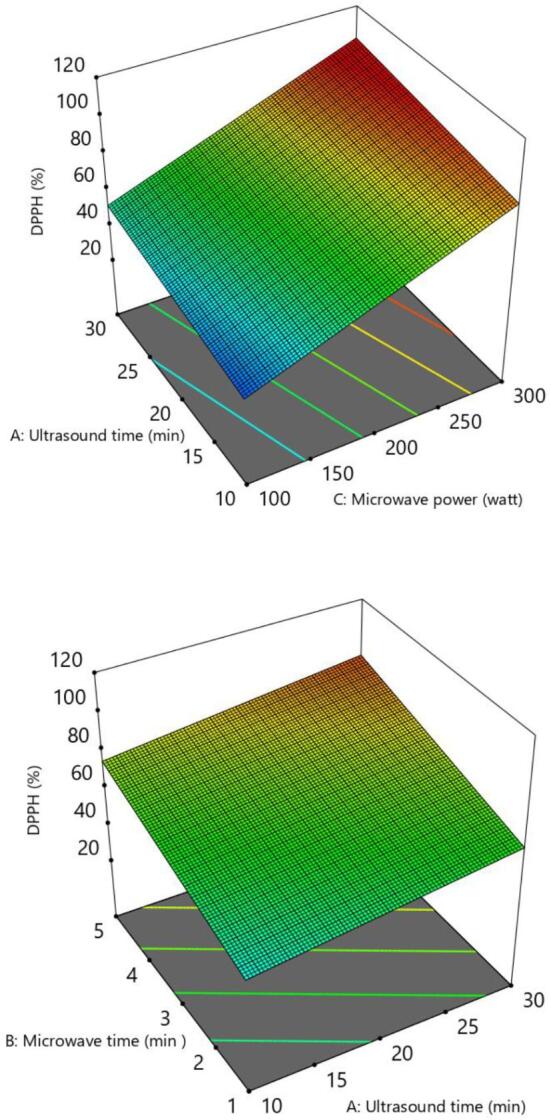
3D Surface plots of DPPH.

### 3.5 The effect of ultrasound and microwave processing parameters on sensory properties of Inula *viscosa* (L.) teas

The quality of Inula *viscosa* (L.) teas were reflected in the 4 major characteristics of color, odor, aroma, and overall acceptability. Fig. 3 shows that color was found darkest in samples 30–5-100 and 10–3-300, by an overall score of 4.6 out of 5. Panelists scored the samples of 30–3-300 and 20–1-100 as the lightest. Consistent with the panelists' evaluations, the samples with the highest and lowest L values were 10–3-300 and 30–3-300, respectively. Among the tea samples, overall acceptance for 10–1-100 and 10–3-300 were rated significantly higher than other tea samples. There is a positive correlation between color and overall acceptability (r = 0.77). In other words, panelists evaluated that the darkest color of tea was more acceptable than those with a lighter color.

It was observed that phenolic compounds were key elements and determined the taste of green tea [28]. 30–5-300 had the strongest aroma score, which was probably related to phenolic compounds in tea, the concentration of which increased during ultrasound treatment. Similarly, the samples had higher TPC and were evaluated as most aromatic by panelists. The correlation between TPC and aroma was calculated as 0.91. Sensory characteristics of teas indicated that the odor was nearly equal for all samples and ranged from 3.8 to 4.5.

## 4 Conclusion

The conditions for optimal drying of Inula *viscosa* (L.) leaves were investigated using ultrasound pretreated microwave drying procedures. Three independent variables (ultrasound pretreatment time, microwave time, and microwave power) that give an optimum value for antioxidant activities optimized by response surface methodology (RSM) based on a user-defined design. The linear models obtained by RSM were accurate and reliable, in which R^2^ and adjusted R^2^ were more than 0.85 with a non-significant lack of fit at p greater than 0.05. Samples dried at 100 and 180 Watts had higher chlorophyll and carotenoid levels than those dried at 300 Watts. When Inula *viscosa* (L.) leaves were treated to 30 min of sonication, the greatest DPPH and TPC levels were discovered. Microwave technology combined with ultrasound pretreatment was used in this work to dry Inula *viscosa* (L.) leaves without compromising their antioxidant and qualitative qualities. The process parameters used will aid in the implementation of a combination of microwave and ultrasonic drying technologies to retain the plant's quality.

## CRediT authorship contribution statement

**Alev Yüksel Aydar:** Conceptualization, Investigation, Formal analysis, Validation, Data curation, Writing – original draft. **Tuba Aydın:** Investigation, Formal analysis, Methodology, Data curation. **Tuncay Yılmaz:** Conceptualization, Methodology, Data curation, Project administration. **Anjinelyulu Kothakota:** Resources, Validation, Data curation, Writing – original draft. **Socol Claudia Terezia:** Funding acquisition, Methodology, Writing – original draft. **Criste Florin Leontin:** Resources, Visualization, Writing – original draft. **R. Pandiselvam:** Conceptualization, Funding acquisition, Methodology, Writing – review & editing.

## Declaration of Competing Interest

The authors declare that they have no known competing financial interests or personal relationships that could have appeared to influence the work reported in this paper.

## Data Availability

Data will be made available on request.
